# High Performance Organic-Nanostructured Silicon Hybrid Solar Cell with Modified Surface Structure

**DOI:** 10.1186/s11671-018-2703-2

**Published:** 2018-09-12

**Authors:** Xiaoli Duan, Xiaofeng Zhang, Yunfang Zhang

**Affiliations:** 1 School of Chemistry and Materials Science, Ludong University, Yantai, 264025 People’s Republic of China; 2 Department of Science, Jiangsu University of Science and Technology, Zhenjiang, 212003 People’s Republic of China

**Keywords:** Silicon nanowire, Tetramethyl ammonium hydroxide, Surface defect, Hybrid solar cells

## Abstract

Silicon nanowires (SiNWs) with excellent light trapping properties have been widely applied in photovoltaic devices, which provide opportunities for boosting the photons harvested by Si. However, the photoexcited carriers are easily trapped and recombined by high-density surface defects due to higher surface area prolonging to depth of nanowire. In this work, in order to reduce the surface defects and recombination rate of SiNWs, a simple solution process is used to modify the surface structure. Applying the tetramethyl ammonium hydroxide (TMAH) treatment leads to smooth and taper Si NW surface, which improves the open-circuit voltage (*V*_oc_) and fill factor (FF) obviously. Thus, a champion PCE of 14.08% is achieved for the nanostructured Si/PEDOT:PSS hybrid device by 60-s TMAH treatment. It also indicates that TMAH treatment promises a simple and effective method for enhancing Si NW-based devices.

## Background

For the photovoltaic devices, the energy conversion efficiency is directly associated with the photos absorption property, which means the more photo incidences, the larger amount electrons can be generated. Thus, the light trapping properties of photovoltaic have been investigated in many works [1–4]. Silicon nanostructures such as silicon nanowire, nanocone, or pyramid arrays have been widely applied due to excellent antireflection properties, which provide opportunities for boosting the photos harvested by Si [5–9]. These nanostructures can be fabricated by a variety of methods, including metal-assisted etching, vapor-liquid-solid growth, reactive ion etching, and laser fabrication [10, 11]. However, despite of the strong optical enhancement, one problem is high surface recombination, which occurs with the high density of surface defects that are associated with the nanostructure. The increased photo carrier recombination decreases the cell efficiency by reducing device fill factor (FF) and open-circuit voltage (*V*_oc_) [12, 13]. This represents the importance of modifying the surface nanostructures to achieve a high performance nanostructure-based solar cell.

Here, we fabricated poly(3,4-ethylenedioxythiophene):poly(styrenesulfonate) (PEDOT:PSS)/Si hybrid solar cells in nanostructured silicon wafers, with various surface morphologies and areas. The conductive polymer, PEDOT:PSS, causes the depletion layer formed in the Si, due to its suitable work function [14, 15]. When the incident photons are harvested by Si substrate, electron-hole pairs are generated. The photo-generated electron-hole pairs are dissociated in the depletion region. The nanostructures in PEDOT:PSS/Si hybrid cells are more representative because the polymer PEDOT:PSS layer is coated on the textured substrate [16, 17]. The surface area and surface recombination are directly associated with the amount of holes that transferred to the electrodes. Moreover, the implementation of nanostructures in PEDOT:PSS/Si hybrid cells is more challenging because the uniform PEDOT:PSS layer can rarely be conformably coated on the textured substrate due to its polymeric characteristics [18, 19]. PEDOT:PSS and the Si nanostructures are needed to allow polymers to infiltrate and form thin films on the surface.

In this work, we explore the TMAH treatment to modify the surface of Si NW, which is fabricated by metal-assisted etching method. By controlling the etching time, we have developed a novel surface nanostructure, which achieves a balance of light trapping property and surface defects. After reducing the surface defects by polishing silicon surface and diminishing the nanowire, the reflectance value is still low. In addition, the effective minority carrier lifetime has been enhanced a lot. A PEDOT:PSS/Si hybrid device using modified Si nanostructure achieves a power convention efficiency (PCE) of 14.08% with a short-circuit current (*J*_sc_) of 31.53 mA/cm^2^, FF of 0.71, and *V*_oc_ of 0.632 V.

## Methods

### Si Nanostructure Fabrication

The fabrication process of the Si NW is followed by a two-step metal-assisted etching method [20]. The Si substrates (0.05~ 0.1 Ω·cm, 300-μm thick) were cut into 1.5 × 1.5 cm^2^. A mixed solution of AgNO_3_ (1 mM) and HF (0.5 vol%) was used to deposit silver nanoparticles. The deposition time was fixed at 60 s. Then, samples were transferred to an etching solution immediately. The etchant solution contains HF (12.5 vol%) and H_2_O_2_ (3 vol%). Vertically aligned Si NWs were formed by etching silicon in the area without silver nanoparticle coverage. To remove the silver nanoparticles, the silicon nanostructures were immersed in concentrated HNO_3_ for 5 min, followed by a DI water rinse for 3 min. Before the TMAH treatment, we need to remove the thin SiO_2_ layer formed during HNO_3_ treatment. Samples were then etched for various times in TMAH (1 vol%) solution at room temperature to decrease the surface area of the silicon nanostructures.

### PEDOT:PSS/Si Heterojunction Solar Cell

After the nanostructured Si substrates have been prepared, PEDOT:PSS film was spin-coated onto the Si substrate. The PEDOT:PSS contains 1 wt% surfactant Trion X-100 and 5 wt% dimethyl sulphoxide (DMSO) to improve the conductivity [21]. The substrate coated with PEDOT:PSS film was annealed at 125 °C for 15 min to remove the solvent water. Finally, silver and aluminum were deposited onto the front and rear side of the device as electrodes. The active area of the device is defined by a shading mask of 0.8 cm^2^.

### Device Characterization

The high-resolution images of the nanostructures were obtained by scanning electron microscope (SEM) images (Carl Zeiss Suppra, 55). The minority carrier lifetime was mapped with microwave-detected photoconductivity MDP map (Freiberg Instrument GmbH). Reflection spectra were measured by an integrating sphere (Perkin-Elmer Lambda 700). Solar cell characteristics were tested by a solar simulator (Newport, 91160) equipped with a xenon lamp (300 W) and an AM 1.5 filter. The irradiation intensity was 100 mW/cm^2^, which was calibrated by a standard Si solar cell device (Newport, 91150). External quantum efficiency (EQE) was acquired from a setup with Newport monochromator 74125 and power meter 1918 with Si detector 918D.

## Results and Discussion

### Morphology and Optical Characterization of SiNW Substrate by TMAH Treatment

**Fig. 1:**
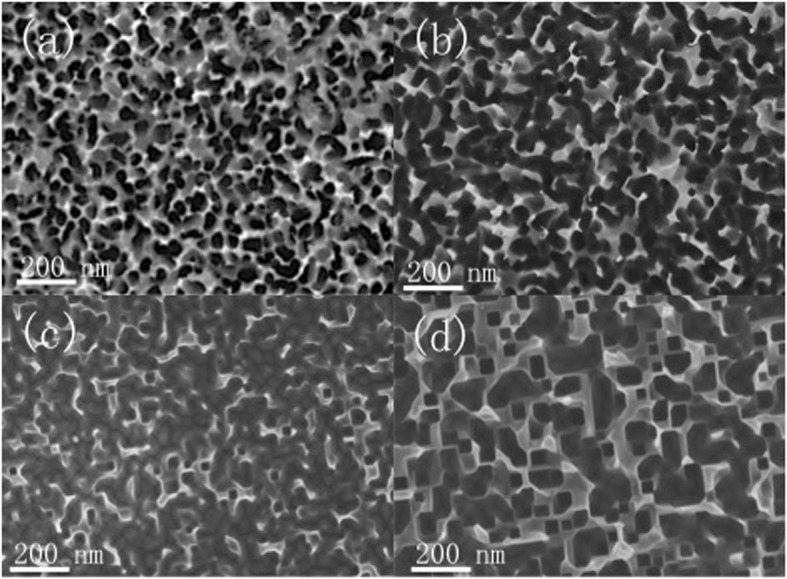
SEM images of different Si nanostructure. **a** As-fabricated Si NW, Si NW with TMAH etching time of **b** 50, **c** 60, and **d** 70 s

The SEM images of fabricated high-density Si nanostructure are shown in Fig. 1a. Si NWs are uniformly distributed on Si wafer with an average wire diameter size of 30 to 50 nm. The nanowires are fabricated from two-step metal-assisted chemical etching [20]. First step, Ag nanoparticles are self-assembled via reduction and oxidation between Ag and Si and, second step, are vertically etched in a mixed etchant solution consisting HF and H_2_O_2_. We can see that the Si NW density is very high, along with large surface area. Figure 1b–d shows the SEM images of Si NW subjected to different anisotropic TMAH etching time from 50 to 70 s. The height is about 120, 100, and 95 nm after etching time of 50, 60, and 70 s, respectively. The etching processing clearly changes the morphology of the nanostructure [22, 23]. Since the concentration of TMAH and etching temperature is constant, with increasing the etching time, more porous SiNWs are etched. We can see that TMAH treatment enable to sparse and taper Si NWs. Moreover, anisotropic TMAH etching forms inversed pyramids at the bottom of nanoholes, which is obvious after 60 s etching. The appearance of inversed pyramids not only dramatically decreases the surface area of nanostructured silicon but also traps the light effectively.

**Fig. 2:**
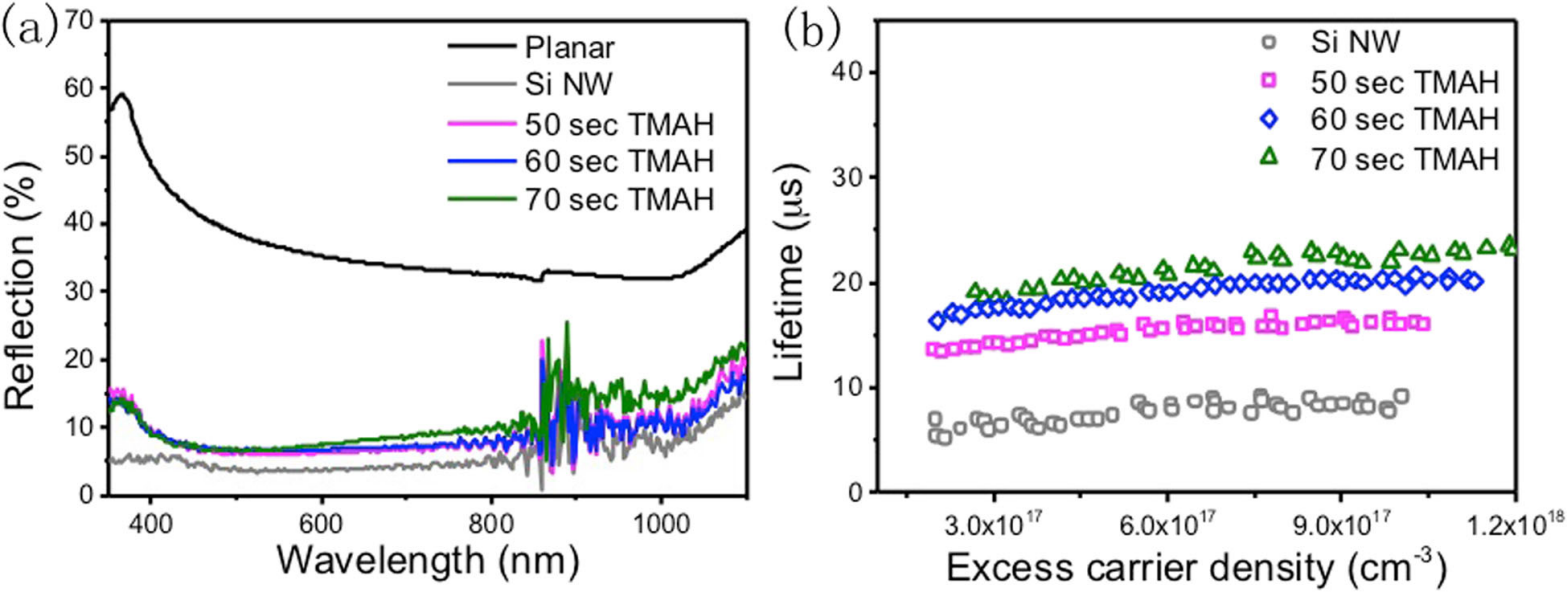
Reflection and minority carrier lifetime characterization of different Si nanostructure. **a** Reflection spectra of various samples: planar Si substrate, Si NW without and with different TMAH time. **b** Injection depended minority carrier lifetime of different samples

In order to evaluate the light-harvesting characteristics of the nanostructures, the reflectance was measured, as shown in Fig. 2a. For the as-fabricated Si NW, the reflectance is relatively low in the wavelength ranging from 300 to 1100 nm. For structures after TMAH treatment, the light trapping property is not as good as the original Si NW structure. However, the average optical reflectance is still low compared with planar Si substrate in all wavelengths. Moreover, the loss of light contributes to reduce the surface defects.

### Surface Recombination of Si NW Substrate by TMAH Treatment

**Fig. 3:**
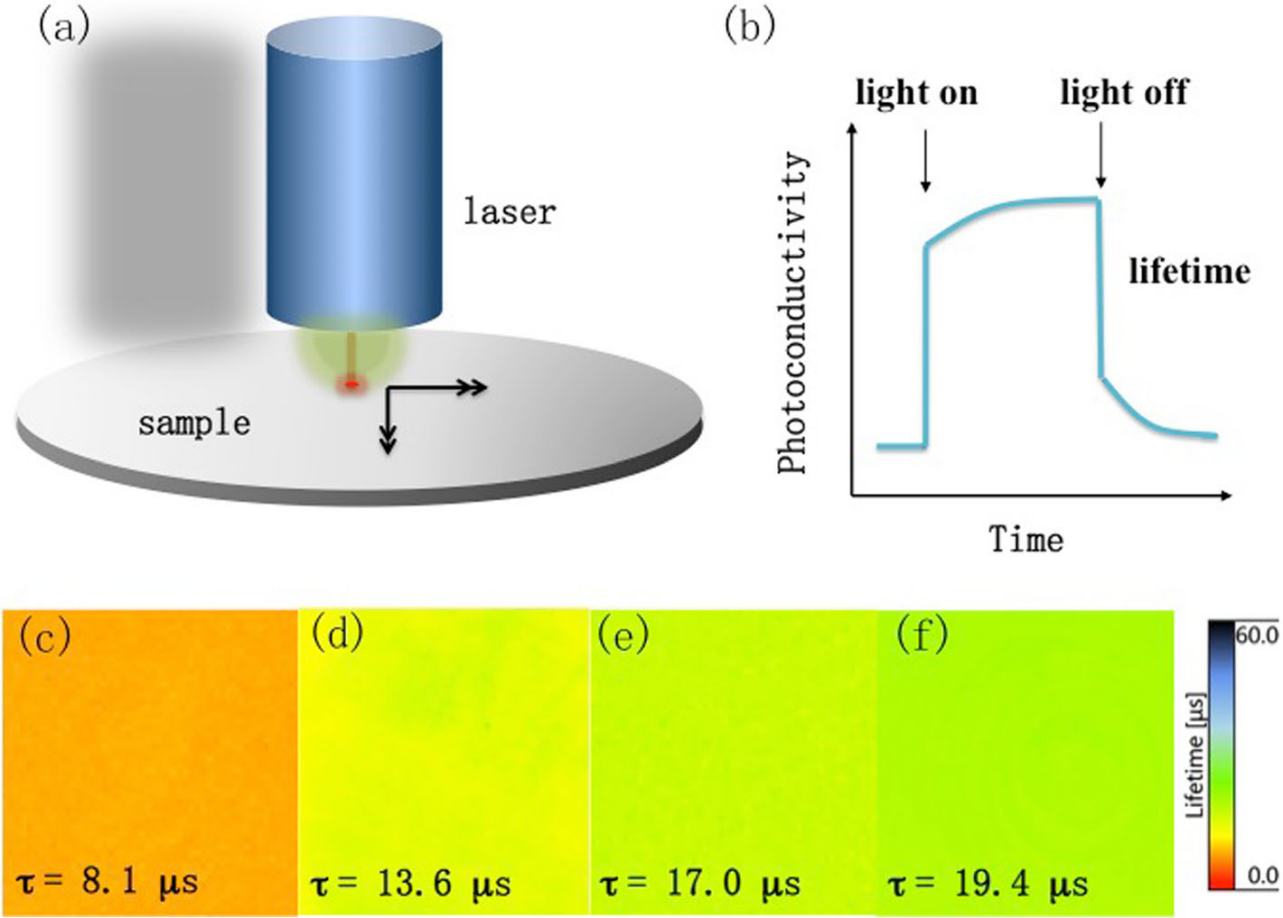
Minority charge carrier lifetime mapping for different Si samples. The schematic diagram of minority charge carrier lifetime: **a** the measurement instruction and **b** the mechanism of carrier lifetime measurement: the photoconductivity, which is closely related to the carrier concentration, is measured by microwave absorption during and after the excitation with a rectangular laser pulse. **c** Si NW without TMAH treatment; Si NW with TMAH treatment for **d** 50, **e** 60, and **f** 70 s. The size of each image was 1.5 × 1.5 cm^2^

To determine the reduction of surface defects, the effective minority carrier lifetime is measured and employed to evaluate recombination mechanisms. Figure 2b shows injection level-dependent effective carrier lifetime (*τ*_eff_) of different etching process samples. The trend of the curve shape is almost the same for these substrates: *τ*_eff_ increases with increasing injection level. At the same injection level, TMAH-treated nanostructured Si substrates exhibit higher *τ*_eff_ than that of Si NW one. Figure 3a, b displays the schematic diagram of the minority charge lifetime measurement. The photoconductivity, which is closely related to the carriers’ concentration, is measured by microwave absorption during and after the excitation with a rectangular laser pulse. Figure 3c–f displays the minority lifetime mapping of different samples at an injection level of 5 × 10^17^ cm^−3^. The average minority carrier lifetime of pristine Si NW substrate is only 8.1 μs, while for the samples with TMAH treatment are 13.6 μs (50 s), 17.0 μs (60 s), and 19.4 μs (70 s).

The minority carrier lifetime of a silicon solar cell follows as the equation below: [24].

$$ \frac{1}{\tau_{\mathrm{eff}}}=\frac{1}{\tau_{\mathrm{bulk}}}+\frac{2S}{W} $$

where *τ* is the effective lifetime, *τ*_bulk_ is the bulk recombination lifetime, *S* is the surface recombination rate, and *W* is the wafer thickness. The increasing minority carrier lifetime indicates lower surface recombination rate since both bulk recombination and thickness were constant for all samples. When the etching time increases, the number of Si NWs decreases, which means less surface defect. As we know, the photo-generated carriers are susceptible to surface recombination loss. With significantly decreased surface area of nanostructures, it is anticipated that the surface recombination processes decrease as well. In turn, in combination with the surface purification and reducing surface area, charge recombination can be dramatically suppressed. For 50, 60, and 70 s etching, the surface area decreases along with smoother surface, result in less surface defect and low recombination rate. If we further increase the TMAH etching time, the silicon nanostructure will diminish and the reflectance value will be much higher.

### Solar Cell Device Performance

**Fig. 4:**
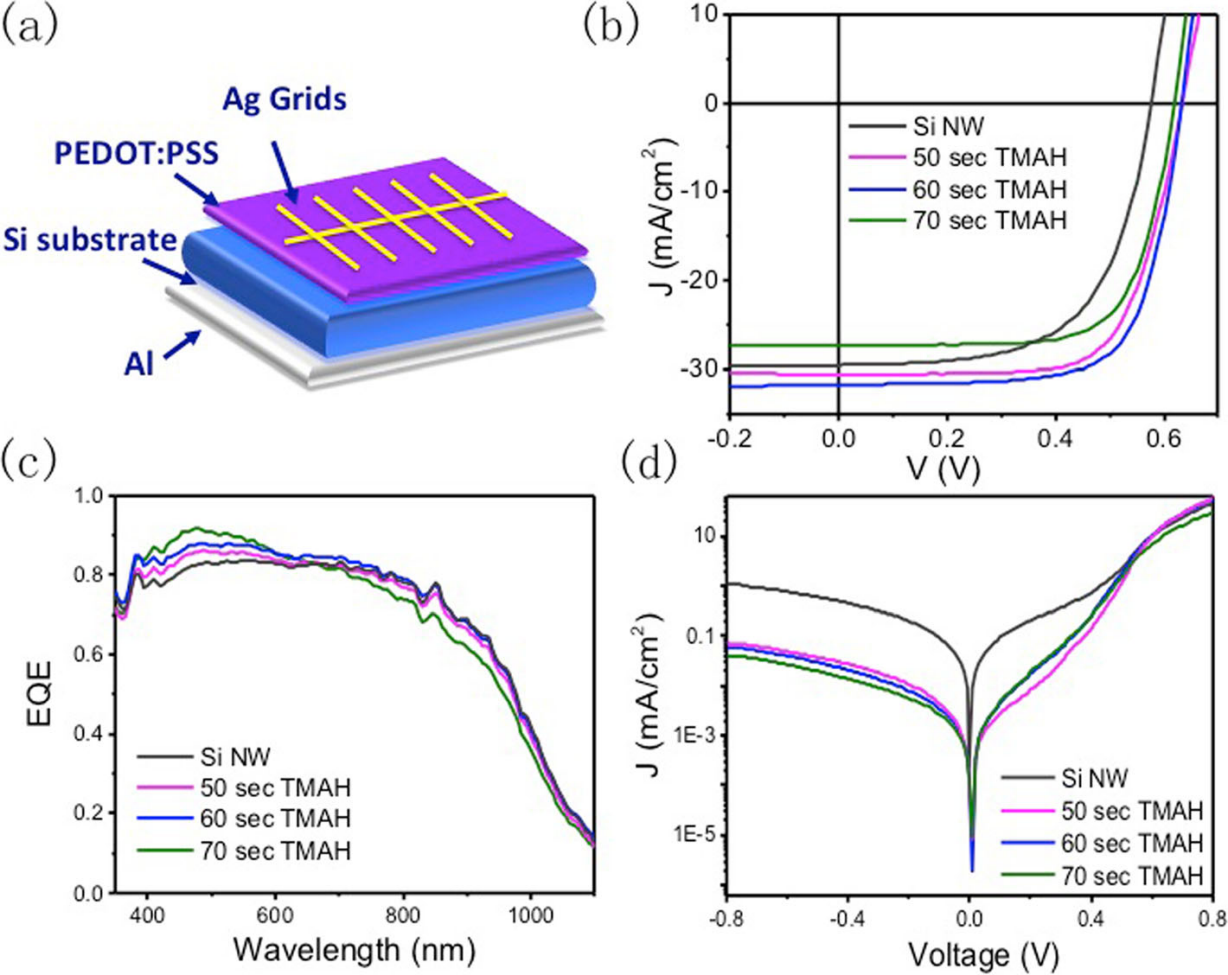
Device performance of the hybrid Si/PEDOT:PSS solar cell: **a** device structure of PEDOT:PSS/Si hybrid solar cell, **b** current density-voltage (J-V) curves of devices based on different nanostructured Si substrate, **c** external quantum efficiency spectra, and **d** J-V curves under dark

The devices structure of PEDOT:PSS/Si hybrid solar cell is shown in Fig. 4a. The performance of devices is summarized in Table 1. Current density versus voltage (J-V) curves of devices with different nanostructured Si substrate is plotted in Fig. 4b. The Si NW-based device exhibits a PCE of 11.02%, *V*_oc_ of 0.584 V, *J*_sc_ of 29.24 mA·cm^−2^, and FF of 0.64. Because of the many defects from the nanostructure, the *V*_oc_ is relatively low. After Si NW polished by TMAH treatment, the device performances improve a lot. For the 50-s etching process, the device yields a PCE of 13.34%, *V*_oc_ of 0.630 V, *J*_sc_ of 30.25 mA·cm^−2^, and FF of 0.70. For the 60-s etching devices, the performance of PCE, *V*_oc_, *J*_sc_, and FF are 14.08%, 0.632 V, 31.53 mA·cm^−2^, and 0.632. And the device of 70-s etching-based substrate exhibits a PCE of 12.16%, *V*_oc_ of 0.628 V, *J*_sc_ of 27.27 mA·cm^−2^, and FF of 0.71. We can find the *V*_oc_ and FF have been enhanced a lot.

**Table 1:** Solar cell efficiency and J–V parameters of the PEDOT:PSS/Si solar cells with different treatment

	V_OC_ (V)	J_SC_ (mA/cm^2^)	FF	PCE (%)
Si NW	0.584	29.24	0.64	11.02
50s	0.630	30.25	0.70	13.34
60s	0.632	31.53	0.71	14.08
70s	0.628	27.27	0.71	12.16

There are two reasons for this enhancement. The first one is that the recombination has been suppressed at the front surface after TMAH polishing treatment, which is testified by the minority lifetime measurement. Moreover, from the EQE measurement shown in Fig. 4c, blue spectral response (400 to 500 nm) of the devices much depended on the substrates structure. With the etching time increase, the EQE in the blue region increases. However, from the reflection spectra, there is a small difference between different nano-structuring processes in this region. So, it is attributed to increased surface recombination processes at the high surface area of the nanostructures. In the large wavelength region, the EQE decreases as the etching time grows. It agrees well with the reflection properties.

**Fig. 5:**
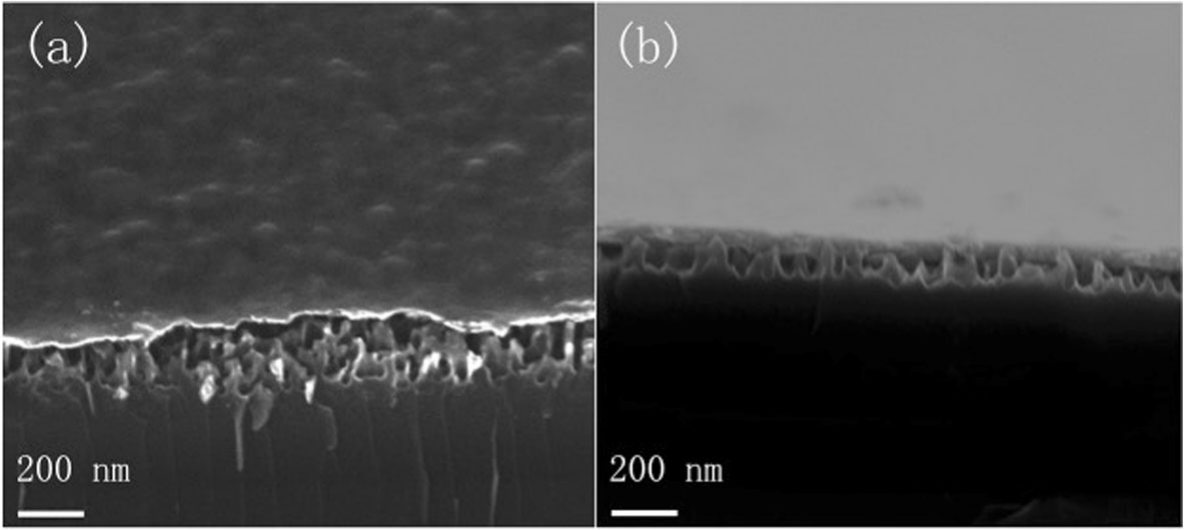
The SEM images of PEDOT:PSS on nanostructured Si substrates: **a** the substrates without TMAH treatment and **b** the substrates with TMAH treatment (60 s)

The second reason is about the contact resistance. As shown in Fig. 5a, the PEDOT:PSS layer can rarely be conformably coated on the random, high-dense Si NW-based substrate. However, when the TMAH treatment has been applied, the nanowires have been tapered and sparse. During the spin coating process, PEDOT:PSS can seep into the gap, shown in Fig. 5b. Moreover, the TMAH treatment induce OH groups over the surface of Si NW, which increase the sticking ability of Si NW and PEDOT:PSS [25, 26]. Thus, the contact area of PEDOT:PSS film and polished-nanostructure substrate is much larger than the Si NW devices. This means the resistance of charge transfer and collection at the front surface can be reduced by the TMAH treatment.

Additionally, the dark J-V curve is shown in Fig. 4d. It was observed that saturation current density (*J*_0_) was suppressed significantly after applying the TMAH treatment. It is commonly accepted that *V*_oc_ strongly depends on the properties at the interface where a low *J*_0_ indicates high-junction quality [27–30]. The decrease of *J*_0_ subsequently favors a more efficient charge separation at the interface and leads to the increase of *V*_oc_, which is consistent with the device performance.

## Conclusions

In conclusion, we have modified the structure of Si substrate for hybrid Si/polymer solar cell with controlled TMAH treatment. This treatment can taper and spare the Si NWs, which reduce the surface area and defects. The minority carrier lifetime is enhanced due to minimizing the surface defect and surface recombination rate. With 60-s TMAH treatment, a PCE of 14.08% has been achieved for the hybrid Si/polymer solar cell. This simple, surface modification process promises an effective method for the nanostructured Si-based photovoltaics.

## Abbreviations

EQE: External quantum efficiency
FF: Fill factor
*J*_sc_: Short-circuit current
PCE: Power convention efficiency
PEDOT:PSS: Poly(3,4-ethylenedioxythiophene):poly(styrenesulfonate)
SEM: Scanning electron microscope
Si NWs: Silicon nanowires
TMAH: Tetramethyl ammonium hydroxide
*V*_oc_: Open-circuit voltage
